# Evidence That Lipopolisaccharide May Contribute to the Cytokine Storm and Cellular Activation in Patients with Visceral Leishmaniasis

**DOI:** 10.1371/journal.pntd.0001198

**Published:** 2011-07-12

**Authors:** Joanna R. Santos-Oliveira, Eduardo G. Regis, Cássia R. B. Leal, Rivaldo V. Cunha, Patrícia T. Bozza, Alda M. Da-Cruz

**Affiliations:** 1 Laboratório Interdisciplinar de Pesquisas Médicas, Instituto Oswaldo Cruz – FIOCRUZ, Rio de Janeiro, Brazil; 2 Laboratório de Pesquisa sobre o Timo, Instituto Oswaldo Cruz – FIOCRUZ, Rio de Janeiro, Brazil; 3 Departamento de Medicina Veterinária, Universidade Federal do Mato Grosso do Sul (UFMS), Mato Grosso do Sul, Brazil; 4 Departamento de Clínica Médica (FAMED), Universidade Federal de Mato Grosso do Sul (UFMS), Mato Grosso do Sul, Brazil; 5 Laboratório de Imunofarmacologia, Plataforma Luminex, Instituto Oswaldo Cruz – FIOCRUZ, Rio de Janeiro, Brazil; Hospital Universitário, Brazil

## Abstract

**Background:**

Visceral leishmaniasis (VL) is characterized by parasite-specific immunosuppression besides an intense pro-inflammatory response. Lipopolisaccharide (LPS) has been implicated in the immune activation of T-cell deficient diseases such as HIV/AIDS and idiopathic lymphocytopenia. The source of LPS is gram-negative bacteria that enter the circulation because of immunological mucosal barrier breakdown. As gut parasitization also occurs in VL, it was hypothesized that LPS may be elevated in leishmaniasis, contributing to cell activation.

**Methodology/Principal Findings:**

Flow cytometry analysis and immunoassays (ELISA and luminex micro-beads system) were used to quantify T-cells and soluble factors. Higher LPS and soluble CD14 levels were observed in active VL in comparison to healthy subjects, indicating that LPS was bioactive; there was a positive correlation between these molecules (r = 0.61;*p*<0.05). Interestingly, LPS was negatively correlated with CD4^+^ (r = −0.71;*p*<0.01) and CD8^+^ T-cells (r = −0.65;*p*<0.05). Moreover, higher levels of activation-associated molecules (HLA-DR, CD38, CD25) were seen on T lymphocytes, which were positively associated with LPS levels. Pro-inflammatory cytokines and macrophage migration inhibitory factor (MIF) were also augmented in VL patients. Consistent with the higher immune activation status, LPS levels were positively correlated with the inflammatory cytokines IL-6 (r = 0.63;*p*<0.05), IL-8 (r = 0.89;*p*<0.05), and MIF (r = 0.64;*p*<0.05). Also, higher plasma intestinal fatty acid binding protein (IFABP) levels were observed in VL patients, which correlated with LPS levels (r = 0.57;*p*<0.05).

**Conclusions/Significance:**

Elevated levels of LPS in VL, in correlation with T-cell activation and elevated pro-inflammatory cytokines and MIF indicate that this bacterial product may contribute to the impairment in immune effector function. The cytokine storm and chronic immune hyperactivation status may contribute to the observed T-cell depletion. LPS probably originates from microbial translocation as suggested by IFABP levels and, along with *Leishmania* antigen-mediated immune suppression, may play a role in the immunopathogenesis of VL. These findings point to possible benefits of antimicrobial prophylaxis in conjunction with anti-*Leishmania* therapy.

## Author Summary

Visceral leishmaniasis (VL) affects organs rich in lymphocytes, being characterized by intense *Leishmania*-induced T-cell depletion and reduction in other hematopoietic cells. In other infectious and non-infectious diseases in which the immune system is affected, such as HIV-AIDS and inflammatory bowel disease, damage to gut-associated lymphocyte tissues occurs, enabling luminal bacteria to enter into the circulation. Lipopolisaccharide (LPS) is a bacterial product that stimulates macrophages, leading to the production of pro-inflammatory cytokines and other soluble factors such as MIF, which in turn activate lymphocytes. Continuous and exaggerated stimulation causes exhaustion of the T-cell compartment, contributing to immunosuppression.

Herein, we show that an increment in LPS plasma levels also occurs in VL; the higher the LPS levels, the lower the TCD4^+^ and TCD8^+^ cell count in the blood. This T-cell depletion may affect the mucosal immune system, which, along with intestinal parasitization by amastigotes, may contribute to gut barrier damage and consequent microbial translocation. LPS levels were correlated with T-cell activation, pro-inflammatory cytokine plasma levels, MIF, and IFABP, showing that a bacterial molecule, probably from luminal origin, not associated with *Leishmania* infection can negatively affect the immune system. These findings points to possible benefits of antimicrobial prophylaxis in conjunction with anti-*Leishmania* therapy.

## Introduction

Visceral leishmaniasis (VL) is a protozoan infection caused in Brazil by *Leishmania* (*Leishmania*) *infantum chagasi.* This disease represents an important worldwide public health problem and affects around 4,000 new cases per year in Brazil [1]. The parasite has a tropism to lymphoid organs, including the bone marrow, spleen, lymph nodes, and liver [2], which explains the immune abnormalities commonly found in affected patients.

VL is a systemic disease with a very complex host-parasite relationship. The parasite affects mainly cells of the macrophage lineage and induces, to some extent, deviations in the production of erythrocytes, platelets, and lymphocytes, consequently generating anemia, thrombocytopenia, and decreased T-cell count [2]. The active phase of VL is characterized by an impairment of the specific effector T-cell response to leishmanial antigens, the absence of a delayed-type hypersensitivity reaction to parasite antigens and a decreased lymphocyte proliferative response, as well as the absence or low levels of interferon (IFN)-γ and interleukin (IL)-2 cytokine production after *in vitro* stimulation of mononuclear cells with *Leishmania* antigens [3], [4], [5]. Elevated type 2 cytokine production has been detected in the serum of VL patients [6]–[8]. In addition, polyclonal activation of B cells and high levels of anti-leishmanial antibody titers are markers of this disease [2]. Restoration of the immune response to *L. chagasi,* as assessed by IFN-γ production and lymphocyte proliferation, has been observed following specific treatment [9], [10].

Interestingly, despite the severe impairment of the *Leishmania*-specific effector immune response, a number of typical markers of chronic activation of the immune system have paradoxically been detected. High levels of pro-inflammatory and anti-inflammatory cytokines have been observed during active disease, but cytokine production returns to normal upon successful chemotherapy [8], [10], [11]. Although there is evidence that components of the parasite play a critical role in determining the immunological abnormalities observed in VL patients, the precise mechanisms underlying these alterations remain unknown [2], [11], [12].

Chronic immune activation has been observed in some infectious and non-infectious diseases, which can contribute to immune suppression and further progression of such illnesses. As an example, infection by human immunodeficiency virus type 1 (HIV-1) induces chronic immune activation and leads to a severe suppression of the T lymphocyte compartment, caused mainly by CD4^+^ T-cell depletion [13]. Early CD4^+^ T-cell depletion in the gastrointestinal tract of HIV-1-infected patients can cause a breach in the integrity of the mucosal immune system, enabling translocation of luminal microbial products into the circulation [14], [15]. Bacterial components such as lipopolisaccharide (LPS) stimulate the adaptive and innate immune system, creating an inflammatory environment that results in a high turnover of T-cell populations, eventually leading to systemic immune impairment [16]. Such a phenomenon can be indirectly detected by measuring LPS plasma levels and has also been observed in conditions such as inflammatory bowel disease [17], idiopathic CD4 lymphocytopenia [18], and prior to hematopoietic transplantation [19].

LPS stimulates mononuclear cells via Toll-like receptor (TLR)-4 and promotes the secretion of a variety of inflammatory soluble factors such as IFN-γ, IL-1, IL-6, tumor necrosis factor (TNF)-α, and macrophage migration inhibitory factor (MIF) [20], [21]. MIF occurs pre-formed and is rapidly released from many cell types in response to different stimuli, including endotoxemia [22]. MIF has also been implicated as a critical factor in the pathogenesis of a variety of inflammatory diseases [23], [24] such as sepsis [25], [26], tuberculosis [27], dengue fever [28], and HIV-1 infection [29].

This enhancement of intestinal permeability which results from mucosal damage and can potentially permits microbial translocation and lead to systemic activation has been assessed by plasma levels of intestinal fatty acid binding protein (IFABP). This cytosolic protein is expressed in the epithelium of small intestine, being released when the cell membrane integrity is compromised [30], such as in patients with abdominal trauma [31] and celiac disease [32].

Our hypothesis was that immunosuppression may also affect the gut mucosal barrier in VL, enabling translocation of microbial products into the systemic circulation. Therefore, we investigated whether plasma LPS levels were increased in VL patients, and the possible association with lymphocyte functions. For these purposes, quantization of T-cell subsets, cell activation status, pro-inflammatory cytokine and MIF, as well as LPS, and IFABP levels were measured in clinical samples from patients with VL.

## Materials and Methods

### Patients and healthy subjects

Ten VL patients were enrolled in this study. Six were males, and the age of the patients ranged between 18 and 60 years (37±19 years). Five patients had active disease without previous anti-*Leishmania* therapy, and 5 patients were evaluated after being cured. Additionally, 2 of the 5 patients with active disease were also subjected to post-therapy evaluation. VL diagnosis was confirmed by identification of amastigotes directly visualized in cells of Giemsa-stained bone marrow smears. Eight healthy subjects (HS) were included as controls; five were males, and the ages ranged between 24 and 32 years (27.2±3 years). They presented a negative proliferative response *in vitro* to antigens of *L. chagasi*. Neither of the VL patients or healthy subjects presented HIV-1 infection, or any other co-morbidities. Patients were treated for VL according to the Brazilian Ministry of Health guidelines [1].

### Ethics statement

This study was approved by the Ethics Committee of the Universidade Federal do Mato Grosso do Sul and by the Ethics Committee of the Fundação Oswaldo Cruz. Written informed consent was obtained from all participants prior to blood collection.

### Immunologic assessments

CD4^+^ and CD8^+^ blood T lymphocytes were quantified by using a BD Tritest® monoclonal antibody specific for CD4, CD8, and CD3 conjugated to fluorescein isothiocyanate (FITC), phycoerythrin (PE), and peridinin chlorophyll protein (PerCP), respectively, and the BDTrue Count reagent kit was used (BD Biosciences, Franklin Lakes, NJ, USA). Samples were acquired using a FACSCalibur (BD Biosciences, USA) and analyzed by Multiset software (BD, USA). The results are expressed as the number of cells/mm^3^.

To evaluate lymphocyte activation, peripheral blood mononuclear cells were obtained by centrifugation over a gradient of Ficoll-Hypaque (Histopaque 1077; Sigma Chemical Company, St. Louis, MO, USA). Cells (10^5^ per tube) were stained with the following monoclonal antibody pairs: anti-CD8 FITC/anti-CD38 PE, anti-CD3 FITC/anti-HLA-DR PE (BD Simultest; BD Biosciences, San Jose, CA, USA), and anti-CD4 PE-Cy5/anti-CD25 PE (BD Biosciences Pharmigen, California, USA). After incubation, the cells were fixed with phosphate-buffered saline plus 1% paraformaldehyde and analyzed by flow cytometry. At least 10,000 events were acquired using a FACSCalibur (BD Biosciences, CA, USA), and phenotypic analysis was carried out by using CellQuest software (BD Biosciences, USA). Results are shown as the percentage of CD3^+^ T, CD4^+^ T, and CD8^+^ T cells expressing HLA-DR, CD25, and CD38 on the membrane surface, respectively. Schematic flow cytometry analysis of T lymphocytes activation-associated molecules is provided in Figure S1 (HLA-DR, CD38, CD25).

### Quantification of LPS, soluble CD14 (sCD14) and IFABP plasma levels

Plasma samples were collected after centrifugation of heparinized venous blood, which was aliquoted and stored at −70°C until the analysis. These samples were diluted in endotoxin-free water, and plasma LPS was quantified by a commercial assay kit (Limulus amebocyte lysate QCL-1000; Cambrex, Milan, Italy) according to the manufacturer’s protocol. The results are expressed as pg/mL and the minimum detected level was 10 pg/mL. The endotoxin levels were measured in leishmanial lysate to exclude any possibility of cross reaction between LPS and *Leishmania* derived LPS-like molecules. Plasma levels of sCD14 were measured using enzyme-linked immunosorbent assay (ELISA) assays (sCD14 Quantikine; R&D Systems, Minneapolis, MN, USA); the results are expressed as ng/mL and the minimum detected level was 125 pg/mL. Plasma IFABP levels were determined using a human highly specific ELISA commercial kit (Duo Set; RD Systems, USA). The results are expressed as pg/mL and the detection limit was 31.2 pg/mL.

### Cytokine measurement

A multiplex biometric immunoassay containing fluorescent dyed microbeads was used for plasma cytokine measurement (Bio-Rad Laboratories, Hercules, CA, USA). The following cytokines were quantified: IFN-γ, TNF, IL-1β, IL-2, IL-4, IL-5, IL-6, IL-8, IL-10, IL-12, IL-13, IL-17, MCP-1 and MIP-1β; cytokine levels were calculated by Luminex technology (Bio-Plex Workstation; Bio-Rad Laboratories, USA). The analysis of data was performed using software provided by the manufacturer (Bio-Rad Laboratories, USA). A range of 0.51–8,000 pg/mL recombinant cytokines was used to establish standard curves and the sensitivity of the assay.

Plasma levels of MIF were measured using an ELISA commercial kit (Duo Set; RD Systems, USA), and the results are expressed as pg/mL; the minimum detected level was 31.2 pg/mL.

### Statistical analysis

The Mann-Whitney test was used to compare data between groups, and correlations between different parameters were analyzed using the non-parametric Spearman’s test. Statistical analysis was confirmed by the Kruskall-Wallis method. Analysis was performed with GraphPad Prism (GraphPad Software version 4.0 for Windows; San Diego, CA, USA). Data were presented as mean ± standard deviation and median. Differences were considered significant when *p*<0.05.

## Results

**Figure 1:**
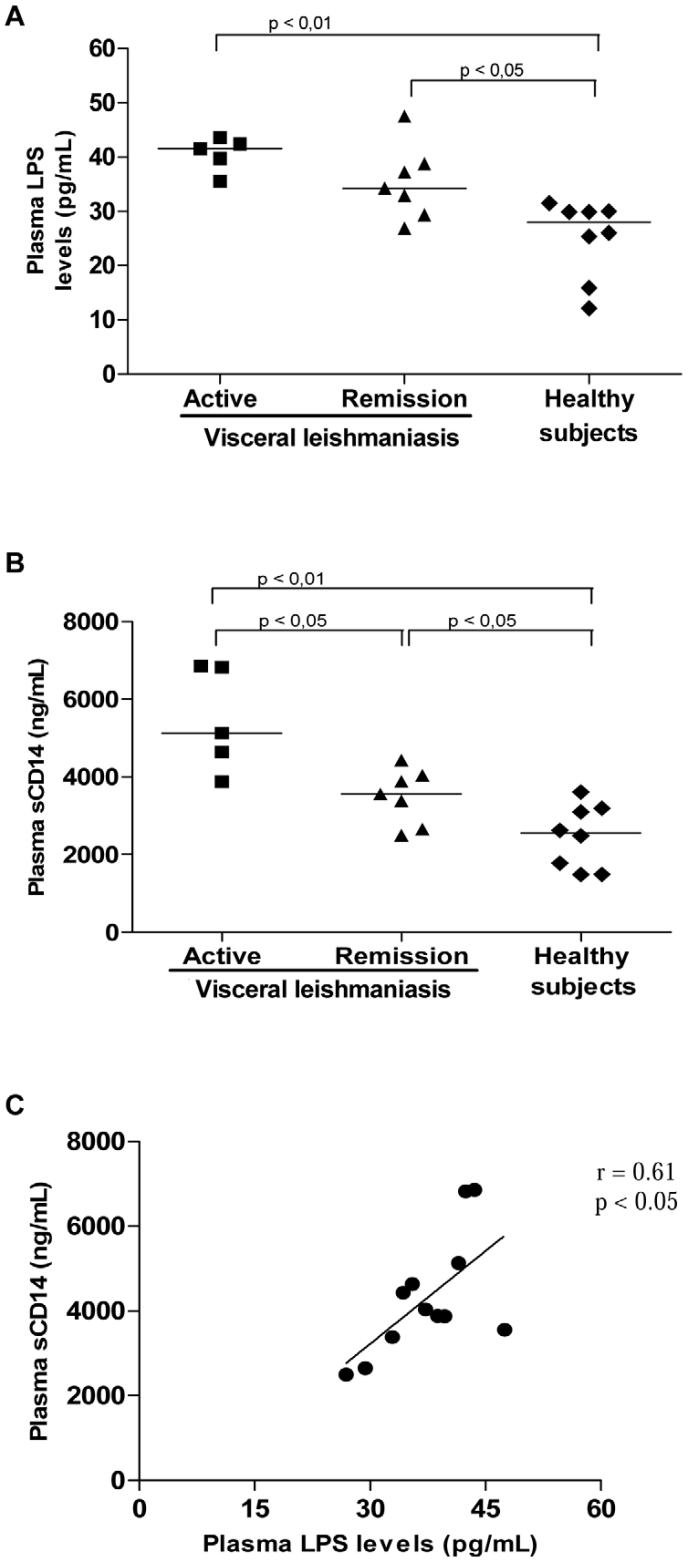
Plasma levels of lipopolysaccharide (LPS) and soluble CD14 in patients with visceral leishmaniasis (VL). Increased LPS (A) and sCD14 (B) levels in VL patients. Correlation between LPS and sCD14 levels in VL patients (C). Active VL patients (solid squares), VL patients in remission (solid triangles), and healthy subjects (solid diamonds). Each point represents 1 subject. The horizontal bars indicate the median value.

Considering that LPS is implicated in the pathogenesis of several immunodeficiency diseases, we examined whether an increment in LPS would also be a prevalent phenomenon in VL. Significantly higher mean levels of LPS were found in the plasma of VL patients (41±3.0 pg/mL, median = 41.5 pg/mL, n = 5) compared to HS (23±8.0 pg/mL, median = 26 pg/mL, n = 8; *p*<0.01, Figure 1A). A tendency towards decreased LPS levels (35±6.0 pg/mL, median = 34.3 pg/mL, n = 7) was seen after therapy, although the levels were still higher than those observed in HS (Figure 1A).

Since it is known that LPS binds to monocytes/macrophages through the CD14 receptor and promotes the release of sCD14 [17], we measured plasma sCD14 levels to indirectly determine whether the LPS in the patients’ blood was biologically active. VL patients presented increased levels of sCD14 (active VL: 5,448±1,359 ng/mL, median = 5,133 ng/mL, n = 5) relative to HS (2,470±815 ng/mL, median = 2,555 ng/mL, n = 8; *p*<0.01). Decreased levels of sCD14 (3,491±712 ng/mL, median = 3,556 ng/mL, n = 7; *p*<0.05) were observed in the remission phase in comparison to before therapy (Figure 1B). In addition, a significant correlation between LPS and sCD14 plasma levels (r = 0.61; *p*<0.05) was found, suggesting that LPS-induced cell stimulation can contribute to sCD14 secretion *in vivo*.

**Figure 2:**
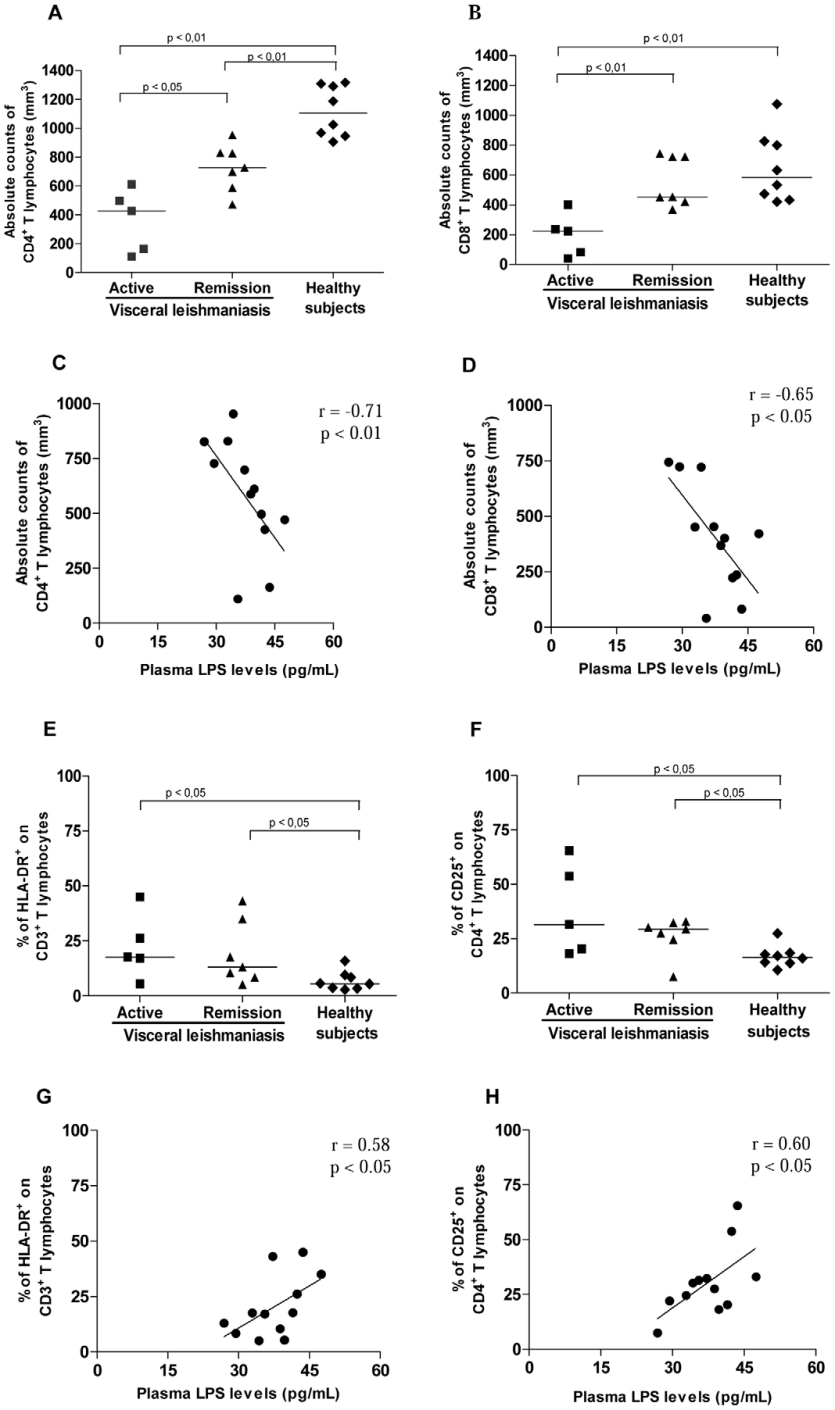
T-cell subset levels and their activation status in patients with visceral leishmaniasis (VL). A. Absolute counts of CD4^+^ T lymphocytes. B. Absolute counts of CD8^+^ T lymphocytes. Correlation between lipopolysaccharide (LPS) plasma levels and the absolute counts of CD4^+^ T lymphocytes (C) and absolute counts of CD8^+^ T lymphocytes (D) in VL patients. E. Levels of HLA-DR^+^ on CD3^+^ T lymphocytes. F. Levels of CD25^+^ on CD4^+^ T lymphocytes. Correlation between LPS plasma levels and percentage of TCD3^+^ cells expressing membrane HLA-DR^+^ (G) and percentage of TCD4^+^ cells expressing membrane CD25^+^ (H) in VL patients. Active VL patients (solid squares), VL patients in remission (solid triangles), and healthy subjects (solid diamonds). Each point represents 1 subject. The horizontal bars indicate the median value.

Further, the repercussions of the LPS increment on lymphocyte effector function were assessed by analyzing T-cell subset counts and cell activation status. Significantly lower numbers of CD4^+^ T cells (361±200 cells/mm^3^, median = 426 cells/mm^3^, n = 5) and CD8^+^ T cells (196±142 cells/mm^3^, median = 223 cells/mm^3^, n = 5) were observed during active disease in comparison to HS (TCD4^+^ = 1,118±175 cells/mm^3^, median = 1,106 cells/mm^3^; TCD8^+^ = 649±230 cells/mm^3^, median = 583 cells/mm^3^, n = 8; *p*<0.05). After treatment, a partial recovery of T-cell counts was observed (TCD4^+^ = 727±160 cells/mm^3^, median = 727 cells/mm^3^; TCD8^+^ = 555±166 cells/mm^3^, median = 456 cells/mm^3^, n = 7; *p*<0.05) in parallel to a satisfactory therapeutic response (Figure 2A and 2B). Active VL patients had a negative proliferative response *in vitro* to *L. chagasi* antigens (data not shown).

Higher mean levels of cellular activation were detected in VL patients, independently of the clinical phase of leishmaniasis, when compared to HS (*p*<0.05, Figure 2E and 2F). However, patients in the active phase had the highest percentage of TCD3^+^HLA-DR^+^ cells (22.3±14%, median = 18%) and TCD4^+^CD25^+^ cells (38±20%, median = 31.5%) when compared to HS (TCD3^+^HLA-DR^+^: 6.8±4.3%, median = 5.5%; TCD4^+^CD25^+^: 16.8±5%, median = 16.5%). Interestingly, the levels of TCD8^+^CD38^+^ cells (active VL = 40±18.5%, median = 40.5%; remission = 45±7.3%, median  = 45.4%) were also augmented in VL patients (HS: 31±4.4%, median = 33%).

To understand whether the elevated LPS levels may be involved in the altered cellular activation status and lymphocyte counts seen in VL patients, correlation analyses were performed. Absolute counts of CD4^+^ (r = −0.71; *p*<0.01) and CD8^+^ T cells (r = −0.65; *p*<0.05) were negatively correlated with LPS (Figure 2C and 2D), showing that the lower T subset numbers were associated with the highest LPS levels during the active phase of leishmaniasis, whereas during clinical remission, the inverse was observed, i.e., the higher T CD4^+^ counts were associated with the lowest LPS levels. Additionally, a significant positive correlation between plasma LPS levels and the frequency of cells with an activated phenotype, i.e., CD3^+^HLA-DR^+^ (r = 0.58; *p*<0.05) and CD4^+^CD25^+^ (r = 0.60; *p*<0.05) T cells, was also found (Figure 2G and 2H).

Another well described consequence of immune activation by LPS is the secretion of pro-inflammatory molecules. To understand whether this process also occurs in VL patients, cytokine concentrations were measured in the plasma of these patients. Similar to T-cell activation, plasma levels of pro-inflammatory cytokines were also elevated in VL patients, not only during active disease, but also up to 6 months after the end of therapy relative to HS (Table 1).

**Table 1:** Pro-inflammatory cytokine levels in the plasma of patients with visceral leishmaniasis (VL).

Cytokinespg/mL	Active VLpatients(n = 5)	VL patientsin remission(n = 7)	Healthysubjects(n = 8)	*p* value*	*p* value**
IFN-γ	1,143(454–4,446)	783(367–3,482)	19(5.3–145)	0.010	0.0010
TNF	143.5(37.7–555)	100(57.3–589)	2(1.5–8.5)	0.001	0.0006
IL-2	35.3(27–38.5)	62(27.6–435)	6(1–9)	0.001	0.0003
IL-6	416(211–557)	49(30–115)	1(0.3–2.5)	0.004	0.0007
IL-8	8,000(1,887–13,578)	4,325(427–8,988)	2.5(1.5–3.0)	0.008	0.0050
IL-1β	9.7(4–226)	7.5(2–138)	0.5(0.4–0.6)	0.001	0.0010
IL-17	288(102–398)	193.5(131–627)	2(2–16)	0.002	0.0006
IL-4	42.3(29.2–54.7)	35.0(23.7–59.1)	5.0(5–12.5)	0.007	0.0020
IL-5	9.4(3.0–143)	2.9(2.6–8.1)	0.4(0.1–1.8)	0.030	0.0300
IL-10	13(8.7–54.8)	12.6(6.0–66)	1.0(0.1–1.6)	0.001	0.0003
IL-12	16.6(10.5–27)	34.0(20.9–44.8)	1.0(0.8–1.4)	0.004	0.0010
IL-13	5.8(3.8–33)	8.3(3.0–12)	1.1(0.7–1.5)	0.001	0.0020
MCP-1	538(285.7–606)	402(327–966.8)	52(31–95.8)	0.004	0.0003
MIP-1β	730.3(418–3,363)	571.5(426–1,111)	14.3(0.1–50)	0.001	0.0010

IFN: interferon, IL: interleukin, TNF: tumor necrosis factor, MCP1: monocyte chemoattractant protein-1, MIP: macrophage inflammatory protein-1β. Statistical analysis was performed using the Mann-Whitney test (*,**) and confirmed by the Kruskall-Wallis method.

Data are presented as median (interquartile range).

*Between active VL patients and healthy subjects.

**Between VL patients in remission and healthy subjects.

**Figure 3:**
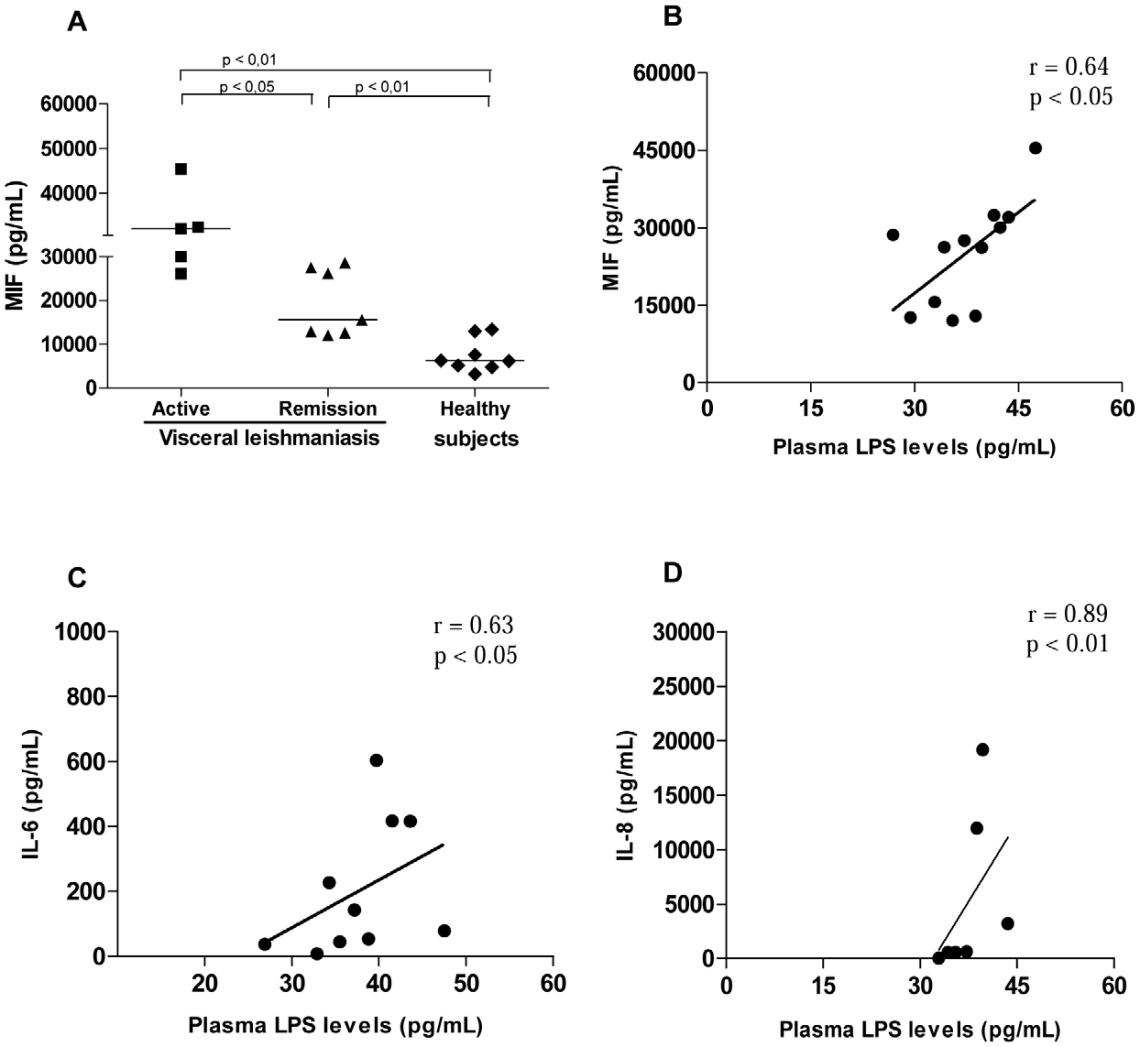
Plasma cytokine levels in visceral leishmaniasis (VL) patients and their association with lipopolysaccharide (LPS) levels. A. Plasma macrophage migration inhibitory factor (MIF) levels in VL patients. Correlation between LPS plasma levels and MIF production (B), IL-6 production (C), and IL-8 production (D) in VL patients. Active VL patients (solid squares), VL patients in remission (solid triangles), and healthy subjects (solid diamonds). Each point represents 1 subject. The horizontal bars indicate the median value.

As LPS also stimulates the secretion of MIF, this molecule was also investigated. MIF was significantly elevated in active VL (33,180±7,273 pg/mL; median = 32,000 pg/mL, n = 5) when compared either with patients during clinical remission (19,348±7,690 pg/mL; median = 15,600 pg/mL, n = 7) or with HS (7,400±3,763 pg/mL; median = 6,238 pg/mL, n = 8; Figure 3A). Even in the remission phase, MIF levels were still increased in comparison to HS (*p*<0.05). Consistent with the higher immune activation status, plasma LPS levels were positively correlated with the inflammatory cytokines IL-6 (r = 0.63; *p*<0.05), IL-8 (r = 0.89; *p*<0.05), and MIF (r = 0.64; *p*<0.05) (Figure 3B, C, and D). The augmented MIF secretion, which correlated with LPS levels, may contribute to the enhanced LPS stimulation in these patients, due to the ability of MIF to up-regulate the cellular expression of TLR-4 [22].

**Figure 4:**
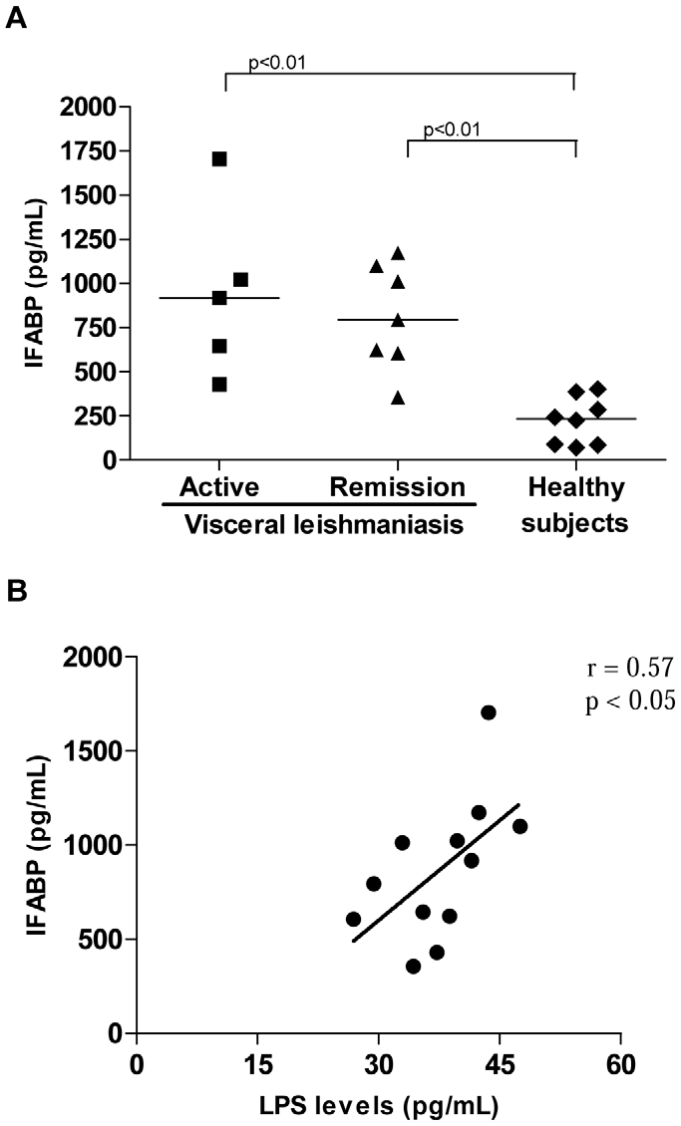
Plasma levels of intestinal fatty acid binding protein (IFABP) in visceral leishmaniasis patients. A. Elevated IFABP levels in VL patients. (B) Correlation between IFABP and LPS levels in VL patients. Active VL patients (solid squares), VL patients in remission (solid triangles), and healthy subjects (solid diamonds). Each point represents 1 subject. The horizontal bars indicate the median value.

Finally, we assessed the plasmatic IFABP levels, since this protein is released into the circulation on intestinal cell injury. VL patients showed higher levels of IFABP (944±484 pg/mL; median = 918 pg/mL, n = 5) in comparison to HS (223.4±133 pg/mL; median = 234 pg/mL, n = 8; p<0.05, Figure 4A). The highest plasma IFABP values were observed during active disease, while remission VL patients presented decreased levels (809±299 pg/mL; median = 794 pg/mL, n = 7). A positive correlation was verified between IFABP and LPS levels (r = 0.57; *p*<0.05), which may suggest some intestinal damage (Figure 4B).

## Discussion

Several circulating molecules that act as soluble receptors for IL-2 [33], immunocomplexes, and lipoproteins [34] have been implicated in VL pathogenesis, but have not been proven to mediate immunosuppressive activity. In this study, it was demonstrated that LPS levels were elevated in VL patients in association with T-cell depletion, lymphocyte activation, and pro-inflammatory cytokine elevation, providing evidence that this factor may play a role in VL immunopathogenesis.

The elevated levels of plasma LPS detected in VL patients indicate that this substance may have a systemic biological effect. It is known that LPS acts via the TLR-4/CD14 receptor complex, leading to sCD14 secretion by monocytes/macrophages [17]. Thus, the elevated levels of sCD14 observed in VL patients in this study indicate that LPS was bioactive in these patients. Furthermore, LPS was positively correlated with sCD14 levels, reinforcing the idea that this microbial product could also contribute to cell activation mechanisms in VL.

Although suppression of the immune response is a hallmark mechanism associated with the development of VL, VL patients present with systemic immune activation as a consequence of pro-inflammatory cytokine release [8], [11], [35], [36], [37]. In this study, it was verified that T lymphocytes up-regulated surface HLA-DR, CD38, and CD25 levels, independently of the clinical phase of VL. These results show that T lymphocytes are also activated in VL, as has been demonstrated in other immunosuppressive diseases such as HIV/AIDS [13], [38]. On the other hand, high lymphocyte activation can lead to death by apoptosis, thereby reducing the number of these cells [2], [11], [12]. In fact, the lowest counts of CD4^+^ and CD8^+^ T cells were observed in patients with active VL, whereas recovery was marked by an increase in the number of these T-cells, which is probably related to the development of an effector response [8], [9], [37]. However, inasmuch as increased levels of these T-cell subsets has been observed during clinical remission, i.e., after successful therapy, these cell counts were still lower in VL patients than in HS, as previously demonstrated [8], [37].

Interestingly, LPS levels in VL patients were negatively correlated with the absolute T CD4^+^ and T CD8^+^ cells counts, showing that higher LPS levels were associated with a lower number of T-cell subsets. In addition, a positive correlation between *ex vivo* cellular activation, represented by HLA-DR and CD25 molecules in T-cells, and plasma LPS levels was found. Firstly, considering that LPS stimulates macrophages to release pro-inflammatory factors such as IL-8, IL-1β, TNF-α, IL-6, and MIF [39], which in turn activate T-cells, it is conceivable that the presence of LPS in the blood stream may contribute to the compromised T-lymphocyte effector function in VL. Secondly, it may be hypothesized that, along with leishmanial antigens, LPS may also contribute to the elevated cellular immune activation in VL. In turn, this activation status can indirectly contribute to the impairment of T cell counts by a mechanism of cell death due to intense stimulation, as has been suggested in other infectious or non-infectious diseases [16], [17]. Even considering the negative correlation results between T cell counts and LPS levels, the approach utilized in this study do not allow a definitive conclusion regarding the causality between these parameters.

The VL patients studied here presented with a cytokine storm characterized by increased levels of pro-inflammatory (IFN-γ, TNF-α, IL-2, IL-6, IL-12, IL-17, and MIF), anti-inflammatory (IL-4, IL-5, and IL-13), and regulatory cytokines (IL-10) and chemokines (IL-8, MCP, and MIP-1β). These findings are similar to those for sepsis [26] and severe dengue fever [40]. Our results also reinforce early data in which a mixed cytokine profile was observed during VL disease [7], [8], [10], [41], [42]. The factors that lead to this systemic activation are still largely unknown. Our data suggest that LPS may play a role in this effect.

It is interesting to note that LPS levels were positively correlated with MIF, a pleiotropic cytokine released after cell exposure to microbial products [39] or to pro-inflammatory cytokines [42]. It is known that MIF up-regulates the expression of TLR-4, the signal transducer molecule of the LPS receptor [20], leading to the production of inflammatory cytokines such as IL-6 and IL-8 [39], [43]. Therefore, MIF production may contribute to the inflammatory milieu in VL patients and the enhancement in the susceptibility to LPS stimulation. As observed for the frequency of T-cells with an activated phenotype, IL-6 and IL-8 levels were also correlated with LPS. In septic patients, high MIF levels have been considered indicative of poor outcome, highlighting the potential therapeutic benefits of MIF neutralization [26]. Our results show that LPS may be contributing not only to the activation of circulating cells, but also to the inflammatory environment observed in VL patients, which may worsen the prognosis. Although LPS levels were lower after VL treatment, it remained higher than those in HS, as also observed for the other immunological parameters investigated. These results indicate that, despite the return of good general clinical status, the reestablishment of immune response homeostasis takes longer to occur. Alternatively, we should considerate that elevated LPS levels could be due to reduced LPS clearance by the kidneys.

The source of the microbial product LPS in the blood stream of VL patients needs to be elucidated. Although, bacterial infections are common complications in VL, none of the patients included in this study presented clinical evidence of such infection. Considering that systemic T-cell depletion can also affect gut-associated lymphoid tissues, as has been suggested for other immunosuppressive pathologies [18], one can suggest the hypothesis that microbial translocation into the blood circulation may also occur in VL. More importantly, gut damage has been extensively documented in VL patients [44]–[46], and cells are heavily parasitized by *Leishmania* amastigotes due to parazitation of mucosal cells, causing frequent diarrhea [45] that is likely to affect the function of this innate barrier. The elevated levels of IFABP, an important plasma marker of gut injury, indicate that intestinal permeability is increased in these patients. Thus, we believe that a vicious circle can be occurring in VL patients, in which systemic inflammation and cellular activation of VL *per se* [7], [8], [11], [41] contributes to gut damage, leading to microbial translocation which in turn increase or sustain the systemic activation status. The phenomenon of microbial translocation has previously been described in graft-versus-host disease [19], inflammatory bowel disease [17], and idiopathic lymphocytopenia [18]. In the context of infectious diseases, microbial translocation demonstrated in HIV-1 infection is associated with LPS-induced systemic immune activation, which indirectly affects the progression toward AIDS [16].

In summary, the present data show that LPS is elevated in VL and possibly contributes, along with *Leishmania* antigens, to the cytokine storm and immune activation status in this pathology. In this context, plasma LPS may be a biomarker suitable for assessing VL prognosis. Considering that LPS originates from gram-negative bacteria from the gut and may reach the blood stream after crossing the gut, the use of antimicrobial prophylaxis in conjunction with anti-*Leishmania* therapy may be beneficial for VL patients, as this strategy may reduce LPS-mediated immune activation in VL.

## Supporting Information

Figure S1 (PDF)
