# Supplementary figures and images for: Evidence That Lipopolisaccharide May Contribute to the Cytokine Storm and Cellular Activation in Patients with Visceral Leishmaniasis
Source: PLoS Negl Trop Dis. 2011 Jul 12;5(7):e1198. doi: 10.1371/journal.pntd.0001198 (PMC3134430; doi:10.1371/journal.pntd.0001198)

A

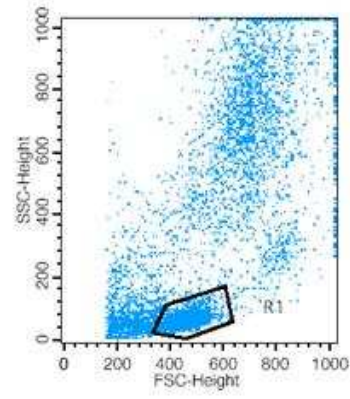

B

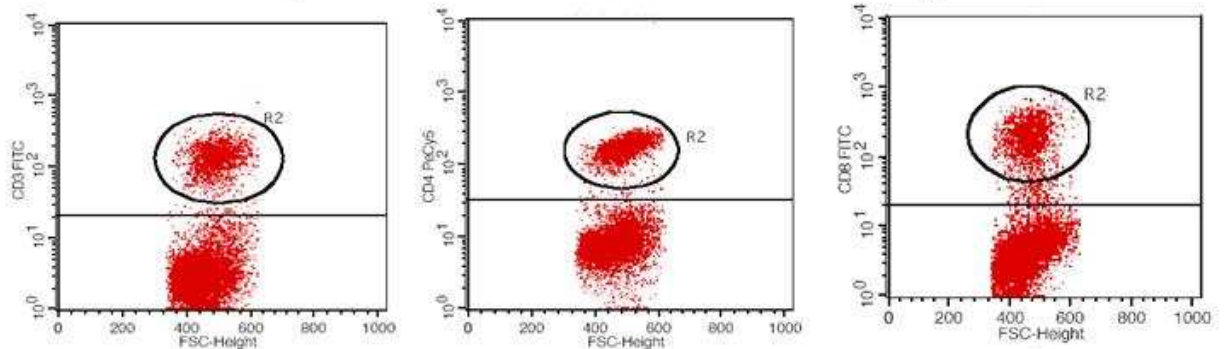

C

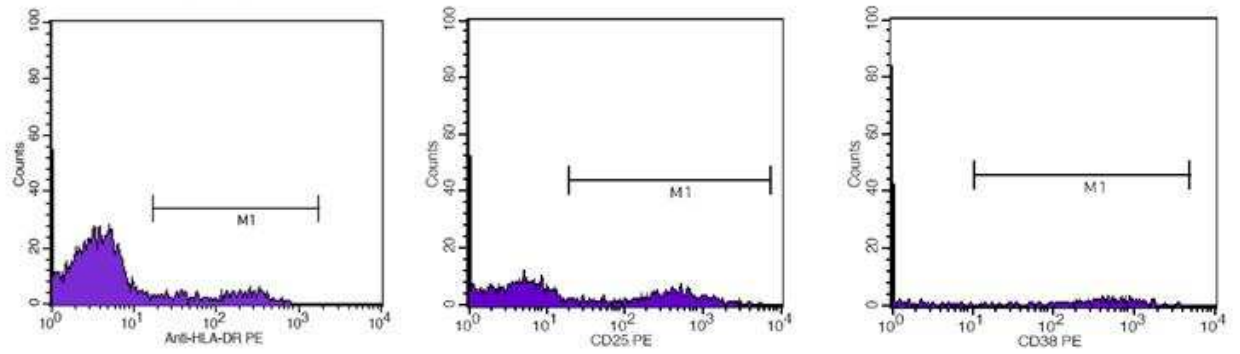

Supplement: Figure S1 — (PDF) [file pntd.0001198.s001.pdf]
